# A 3D Scan Model and Thermal Image Data Fusion Algorithms for 3D Thermography in Medicine

**DOI:** 10.1155/2017/5134021

**Published:** 2017-11-08

**Authors:** Adam Chromy, Ondrej Klima

**Affiliations:** ^1^Faculty of Electrical Engineering and Communication, Brno University of Technology, Brno, Czech Republic; ^2^Faculty of Information Technology, IT4Innovations Centre of Excellence, Brno University of Technology, Brno, Czech Republic

## Abstract

**Objectives:**

At present, medical thermal imaging is still considered a mere qualitative tool enabling us to distinguish between but lacking the ability to quantify the physiological and nonphysiological states of the body. Such a capability would, however, facilitate solving the problem of medical quantification, whose presence currently manifests itself within the entire healthcare system.

**Methods:**

A generally applicable method to enhance captured 3D spatial data carrying temperature-related information is presented; in this context, all equations required for other data fusions are derived. The method can be utilized for high-density point clouds or detailed meshes at a high resolution but is conveniently usable in large objects with sparse points.

**Results:**

The benefits of the approach are experimentally demonstrated on 3D thermal scans of injured subjects. We obtained diagnostic information inaccessible via traditional methods.

**Conclusion:**

Using a 3D model and thermal image data fusion allows the quantification of inflammation, facilitating more precise injury and illness diagnostics or monitoring. The technique offers a wide application potential in medicine and multiple technological domains, including electrical and mechanical engineering.

## 1. Introduction

In recent years, the availability of thermal imagers has moved from expensive, bulky, and cumbersome systems to affordable and practical solutions [1]. Applicable sensors and filters have been developed to such an extent that thermal cameras can be found already in smartphones at prices up to 700 EUR [2]. Due to such rapid progress, thermal imaging is being practically employed on an everyday basis also in fields and disciplines where it previously functioned as an instrument convenient exclusively for research purposes.

In the given context, a typical target field is, for example, medicine: digital medical thermal imaging (DMTI), a modality of medical imaging to monitor surface skin temperature, has been evolving over the last 50 years to contribute towards improving evidence-based diagnosis and facilitating the early detection of diseases.

Within medicine, current applications of the technique are to be sought primarily within clinical procedures centred on assessing and monitoring peripheral vascular, neurological, and musculoskeletal conditions within multiple medical subdisciplines, including cardiology, dermatology, dentistry, obstetrics, oncology, physiotherapy, public health, surgery, and veterinary medicine, and the investigation of chronic and occupational diseases [3].

Although the 2D thermal imaging is able to quantify the temperature of the individual pixels of the image, the DMTI is still considered a mere *qualitative tool*, enabling us to distinguish between the physiological and nonphysiological states of the body but lacking the ability to *quantify* them [3, 4]. This is due to three main drawbacks of DMTI: almost impossible definition of the region of interest (ROI) in a thermal image due to lack of recognizable clearly bounded thermal features in the image, distortions caused by transforming 3D world to 2D representation, and dependence of the thermogram on the view of the camera. The first drawback makes measurements of average ROI temperature impossible, the same as differential measurements between two ROIs, what are the main methods of medical thermal quantification (the single thermal values are not used for quantification, since the surface body temperature is influenced by previous physical activity, stress, etc. From this reason, the comparison between average temperatures of the reference area and ROI shall be used). The second drawback disallows also measurements of an affected area, and the third one disqualifies evaluation of changes during the time.

Nearly all types of injury, together with many diseases or pathological changes, are characterized by an increased blood flow and a stronger cellular metabolic rate in the affected region; the two aspects cause a local increase of temperature *proportional* to the phenomenon [5]. This proportional dependence predetermines that quantification via DMTI should be possible.

Another rapidly advancing technology consists in 3D scanning. 3D surface models find increasingly intensive use in situations where an object must be preserved in a permanent, time-invariant state. In such cases, colour-covered 3D models seem to constitute the best modality [6]. Further, at present, object cloning could also be named as a dynamically growing domain. Multiple types of 3D printers are available on the market, and each of them requires a tool to build the 3D model to be printed [7]. Finally, computer-based 3D models are, due to their plasticity, becoming ever more favoured in the visualization of objects characterized by good visibility but also a small size of major details, which then have to be enlarged plastically [8]. These solutions and applications, by definition, exhibit a strong potential to be used also in healthcare too.

If combined, the two above-outlined, state-of-the-art technologies could yield a volume of new information even higher than that obtainable through their separate use. Such data fusion would subsequently enable us to address some of the long-term challenges to be resolved within diverse medical sectors.

One such problem lies in *medical quantification*, an issue encompassing the entire healthcare system: evaluation methods excessively inaccurate, insensitive, or subjective embody a principal drawback affecting, for example, dermatology, traumatology, physiotherapy, and forensic sciences.

In dermatology, the degree of objectivity in evaluating disease severity and the extent of lesions is still insufficient, due in particular to the lack of reliable in vivo quantification methods to assess the concrete region of interest only.

Traumatology and forensic sciences suffer from the absence of methods to cope with the quantification of bruise severity, often through time.

In physiotherapy, techniques are unavailable for detecting early tiny changes in the body volume, a possible symptom of an emerging disease. It is also rather difficult to distinguish between physiological (e.g., muscle growth) and nonphysiological (e.g., swelling) changes, and the impact of treatment procedures on a disease cannot be quantified smoothly, because the current evaluation methods are mostly based on subjective perception, health surveys and related forms, or low-resolution scoring systems exhibiting poor interobserver correlation.

**Figure 1:**
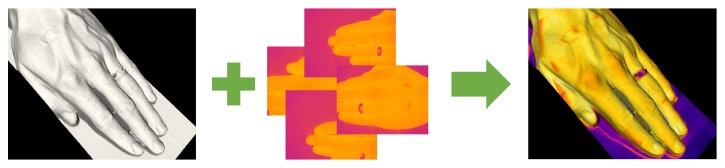
Visualizing the data fusion process: the 3D model from a 3D scanner (left) is combined with the 2D thermal images obtained using a thermal camera (middle) to produce the final 3D thermal model (right).

These and many other issues are solvable using 3D thermal quantification. An effective approach appears to consist in extending a 3D scanner with a thermal imaging sensor and mapping relevant thermal information onto the surface of the 3D model via data fusion algorithms (Figure 1) [9].

Such a combination of sensors generates a multilayered 3D model of the patient's body, containing the temperature at each surface point and embodying an extension of the 3D volume that constitutes the output of a standard 3D scanner. By studying the distribution of the temperatures along the surface of the body, we can then easily localize and, subsequently, quantify the inflammation foci (in the sense of the average temperature gradient in the affected region or its extent). At the following stage, the volume increment caused by swelling can be precisely measured.

Besides inflammation monitoring, merging thermal and spatial data allows several other medical applications. While inflammation increases the local temperature, necrosis leads to its decrease; thus, the device characterized herein can be used in, for example, monitoring diabetic necrotic tissues.

This paper discusses data fusion algorithms to merge a 3D model (captured by any 3D scanner) and thermal images (captured by any thermal imager). In this context, the following section introduces a generally applicable process of combining the thermal and spatial data; importantly, the related significance and usefulness for medical diagnosis are experimentally demonstrated on 3D thermal models of real patients.

## 2. Materials and Methods

The section outlines a procedure for merging 3D data and thermal images. The algorithms introduced below are applicable to a *general* digital 3D model of an object provided by *any* 3D scanner and usable with the *general* thermal data produced by *any* thermal imager. The only requirement is to know the location and orientation of the camera relevant to each captured image. After this condition has been satisfied, the data fusion algorithm is fully automatic and does not require manual assistance.

**Figure 2:**
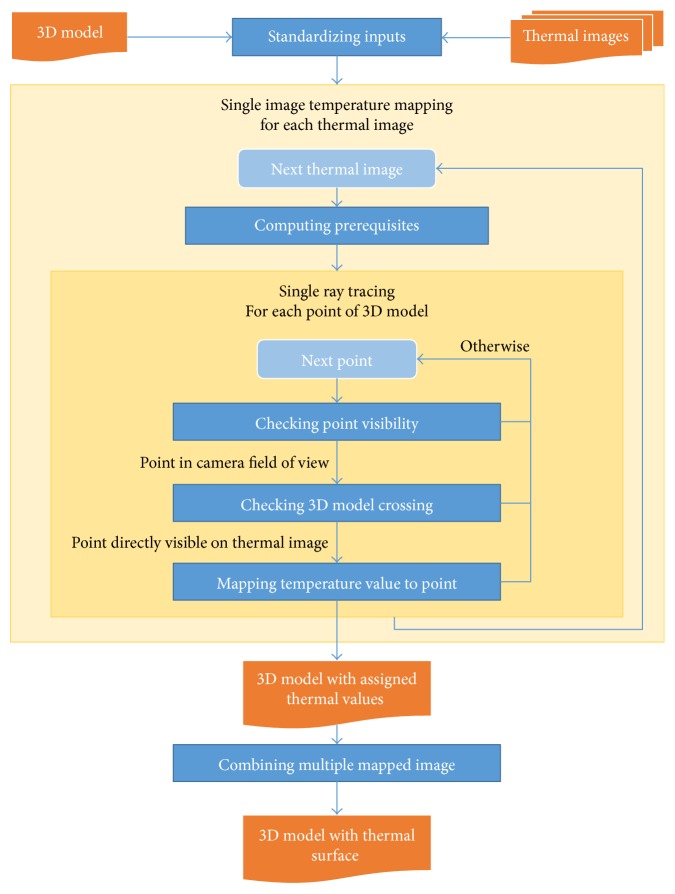
A schematic diagram of the data fusion algorithm.

The entire algorithm is set forth within the diagram in Figure 2, and all the procedures are further explained in the following sections.

### 2.1. Standardizing Inputs

Various 3D scanners provide the output data in diverse, more or less standardized, digital formats. Even though the protocol, structure, and data type vary between the different forms, they share a common feature: the data can be considered a *triangle mesh*, namely, a *set of triangles* defined by three points, with each of these defined by three coordinates in the Cartesian space. The data may assume the shapes of another polygon mesh (e.g., a quadrilateral mesh or a set of quadrilaterals) or an unordered set of points (point cloud) [10]. The first type is easily transferable to triangle mesh because every convex polygon can be divided to a number of triangles [11], and the other one enables conversion to a triangle mesh via any triangulation algorithm [12], for example, a Delaunay triangulation [13].

Thermal imagers also exhibit different file formats; in all cases, however, the thermal data are obtainable as a *2D matrix of scalar temperature values*. Some cameras supply such matrices directly, while others provide coloured images in the bitmap form. In the latter option, the transformation scale between the colour and the temperature values is yielded, whereby the colours can be translated to scalars [14]. In general terms, however, the thermal data are represented as a 2D matrix where each value refers to the temperature of a particular pixel.

The discussion below also presumes no radial and tangential distortion of the image, meaning that the input images shall be already preprocessed according to the intrinsic parameters of the camera.

Proper image alignment is achievable if the following thermal imager parameters are known:

1. Location of the camera focus in space (vector $\vec{T_{from}}$)
2. Camera view direction (unit vector $\vec{T_{\text{to}}}$)
3. Direction defining “up” in the camera image (unit vector $\vec{T_{up}}$, perpendicular to $\vec{T_{\text{to}}}$)
4. Angle of view of the camera in the horizontal (*δ*_H_) and vertical (*δ*_V_) dimensions (in radians)
5. Focal distance of the camera optics (scalar *T*_FD_)
6. Number of values (resolution) in the thermal image along the horizontal (*I*_W_) and vertical (*I*_H_) dimensions.

The first three parameters are usually measured directly using various tracking systems [15] or estimated from scene changes (ICP-based methods, [16]). Parameters 4–6 are mostly known from the technical documentation of the camera; alternatively, they can be acquired through the calibration method published in [17].

It is important to emphasize that these 6 parameters exert a significant influence on proper matching between the thermal images and the 3D model, and we thus need to know them with a high accuracy. The calibration methods relevant to these tasks are characterized in [17–19]. The properly mutually calibrated sensors, providing these 6 parameters with high accuracy and then ensuring correct registration of thermal images onto the 3D model, are assumed in further text.

### 2.2. Computing Prerequisites

**Figure 3:**
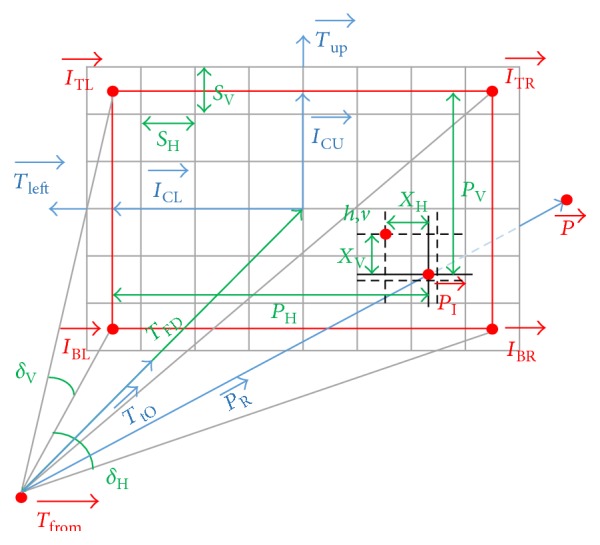
The meaning of the main variables from the thermal image mapping algorithm. Here, the red variables are the positional vectors; the blue ones denote the directional vectors; and the green values represent the scalars.

The computations below are associated with certain prerequisites, which can be computed once per mapped image (Figure 3) in order to keep the algorithm fast.

The position of the thermal image, located in real coordinates and defined by its top-left ($\vec{I_{TL}}$), top-right ($\vec{I_{TR}}$), bottom-left ($\vec{I_{BL}}$), and bottom-right ($\vec{I_{BR}}$) corners, is computable as

(1) $$\begin{array}{c} \vec{I_{TL}}=T_{FD}*\vec{T_{\text{to}}}+\vec{I_{CU}}+\vec{I_{CL}},\\ \vec{I_{TR}}=T_{FD}*\vec{T_{\text{to}}}+\vec{I_{CU}}-\vec{I_{CL}},\\ \vec{I_{BL}}=T_{FD}*\vec{T_{\text{to}}}-\vec{I_{CU}}+\vec{I_{CL}},\\ \vec{I_{BR}}=T_{FD}*\vec{T_{\text{to}}}-\vec{I_{CU}}-\vec{I_{CL}}, \end{array}$$

where the vectors $\vec{I_{CU}}$ and $\vec{I_{CL}}$ point away from centre of the image, upwards or leftwards. Both the vectors are shortened by one half of a pixel size as each pixel represents the average colour on its surface. We then have

(2) $$\begin{array}{c} \;\vec{I_{CU}}=\left(\vec{T_{up}}*\tan\frac{\delta_{V}}{2}*\left|T_{FD}*\vec{T_{\text{to}}}\right|\right)*\left(1-\frac{1}{I_{H}}\right),\\ \vec{I_{CL}}=\left(\vec{T_{left}}*\tan\frac{\delta_{H}}{2}*\left|T_{FD}*\vec{T_{\text{to}}}\right|\right)*\left(1-\frac{1}{I_{W}}\right),\\ \vec{T_{left}}=\mathrm{norm}\left(\vec{T_{up}}\times \vec{T_{\text{to}}}\right). \end{array}$$

The size of a single pixel in the horizontal (*S*_H_) and vertical (*S*_V_) dimensions can be derived as follows:

(3) $$\begin{array}{c} S_{H}=2\frac{\left(\tan\;\left(\delta_{H}/2\right)*\left|T_{FD}*\vec{T_{\text{to}}}\right|\right)}{I_{W}},\\ S_{V}=2\frac{\left(\tan\;\left(\delta_{V}/2\right)*\left|T_{FD}*\vec{T_{\text{to}}}\right|\right)}{I_{H}}. \end{array}$$

### 2.3. Single Image Temperature Mapping

The central concept of the mapping algorithm is to trace the rays between the thermal imager's origin and each point of the scanned 3D model. For each point of the 3D model, the steps to be taken are as shown in the following portion of the article: this single mapping procedure is performed for each thermal image, resulting in the assignment of several thermal values to each point of the 3D model (the number of the thermal values assigned to a single point is given by the number of those images where the particular point is directly visible).

#### 2.3.1. Checking the Point Visibility

In the initial phase, we need to check whether the point ($\vec{P}$) lies within the imager's field of view, namely, if the ray $\vec{P_{R}}=\vec{P}-\vec{T_{from}}$ from the imager's focus to the point intersects the plane in which the thermal image is located (the plane is defined by three arbitrary points from the image corner points $\vec{I_{TL}}$, $\vec{I_{TR}}$, $\vec{I_{BL}}$, and $\vec{I_{BR}}$).

If we find an intersection point ($\vec{P_{I}}$), the algorithm is left to continue; otherwise, we skip the related following steps and continue with step 1 for the next point of the 3D model.

Then, it has to be established whether $\vec{P_{I}}$ lies within the thermal image rectangle. This is true when all the following conditions are satisfied [20]:

(4) $$\begin{array}{c} \mathrm{norm}\left(\vec{I_{BR}}-\vec{I_{BL}}\right)\cdot \mathrm{norm}\left(\;\vec{P_{I}}-\vec{T_{from}}-\vec{I_{BL}}\right)>0,\\ \mathrm{norm}\left(\vec{I_{TR}}-\vec{I_{BR}}\right)\cdot \mathrm{norm}\left(\;\vec{P_{I}}-\vec{T_{from}}-\vec{I_{BR}}\right)>0,\\ \mathrm{norm}\left(\vec{I_{TL}}-\vec{I_{TR}}\right)\cdot \mathrm{norm}\left(\;\vec{P_{I}}-\vec{T_{from}}-\vec{I_{TR}}\right)>0,\\ \mathrm{norm}\left(\vec{I_{BL}}-\vec{I_{TL}}\right)\cdot \mathrm{norm}\left(\;\vec{P_{I}}-\vec{T_{from}}-\vec{I_{TL}}\right)>0. \end{array}$$

If, however, the above items are not fulfilled, we skip again.

#### 2.3.2. Checking the 3D Model Crossing

Satisfying the conditions above does not suffice to determine if a point is *directly* visible, as that point can be hidden behind a part of the 3D model. Thus, we are obliged to check if the ray $\vec{P_{R}}$ intersects the 3D model or not.

The simplest procedure to find the intersection consists in verifying whether the ray $\vec{P_{R}}$ intersects *any* of the triangles which constitute the 3D model. To check the ray-triangle intersection, the algorithm from source [21] is used.

The algorithm iterates throughout all the triangles. When the ray-triangle intersection is located, the iteration stops, and we skip. With all the triangles checked without the intersection found, the point $\vec{P}$ is *directly* visible from $\vec{T_{from}}$, and we continue with the last step; otherwise, the stage is skipped.

#### 2.3.3. Mapping the Temperature Values to the Point

After the direct visibility has been proved, the temperature value for the given point is computed as the linear interpolation between the 4 nearest neighbouring pixels, taking into account the distance from the intersection $\vec{P_{I}}$ to the pixels.

The indices (the horizontal index *h* and vertical index *v*) of the nearest pixel from $\vec{P_{I}}$ in the top-left direction are determined as follows:

(5) $$\begin{array}{c} h=floor\left(\frac{P_{H}}{S_{H}}\right),\\ v=floor\left(\frac{P_{V}}{S_{V}}\right),\\ P_{H}=\left|\mathrm{norm}\left(\vec{I_{BL}}-\vec{I_{TL}}\right)\times \left(\vec{P_{I}}-\vec{T_{from}}-\vec{I_{TL}}\right)\right|,\\ P_{V}=\left|\mathrm{norm}\left(\vec{I_{TL}}-\vec{I_{TR}}\right)\times \left(\vec{P_{I}}-\vec{T_{from}}-\vec{I_{TR}}\right)\right|. \end{array}$$

The distance of the intersection $\vec{P_{I}}$ from this pixel (expressed as a percentage of the pixel size) in the horizontal (*X*_H_) and vertical (*X*_V_) directions is

(6) $$\begin{array}{c} X_{H}=\frac{P_{H}\;\mathrm{mod}\;S_{H}}{S_{H}},\\ X_{V}=\frac{P_{V}\;\mathrm{mod}\;S_{V}}{S_{V}}. \end{array}$$

The temperature *t*_P_ belonging to the point *P* is then interpolated from the temperatures of the neighbouring pixels *t*_*h*,*v*_, *t*_*h*+1,*v*_, *t*_*h*,*v*+1_, and *t*_*h*+1,*v*+1_:

(7) $$\begin{array}{c} t_{P}=interp\left(interp\left(t_{h,v},t_{h,v+1},X_{H}\right),interp\left(t_{h+1,v},t_{h+1,v+1},X_{H}\right),X_{V}\right),\\ interp\left(t_{1},t_{2},d\right)=\left(1-d\right)*\;t_{1}+d*\;t_{2}. \end{array}$$

### 2.4. Combining Multiple Mapped Images

The temperature mapping procedure outlined in the previous section assigns several temperature values to each point visible in the thermal image. If more overlapping thermal images are mapped, then a correspondingly increased count of values is assigned to a single point of the 3D model.

Pursuing the development of medical thermography, we use long-wave infrared thermal imagers (LWIR) to detect the thermal radiation from the scene; such radiation consists of the reflected and the emitted forms [22]. The typical emissivity of a naked human body ranges between 0.93 and 0.96 [23], meaning that the major part of the radiation detected by a thermal imager consists in the emitted form; reflected radiation thus plays a minor role.

Our experiments also show the validity of this claim in that the values belonging to a single point of the 3D model, acquired via the images captured from several different orientations, varied at the sensor noise level only. Thus, the thermal radiation reflection can be considered negligible.

The final point temperature value is thus simply computable as the average temperature from all the values associated with the particular point.

### 2.5. Optimizing the Algorithm Performance

Even though the algorithm to check the ray-triangle intersection [21] is very fast, iterating throughout the entire set of triangles remains significantly slow.

The procedure execution time can be markedly decreased by a hierarchical structure allowing us not to check triangles remote from the ray. The presented algorithm exploits a modification of the octree data structure, facilitating the partitioning of the 3D space by recursive subdivision into eight octants [24, 25].

The minimal rectangular spatial area aligned with the axes into which the model extends is divided into a number of same-sized cubes. The triangles of the 3D model are split to form cubes respecting their relevant locations in the 3D space. To enable the assignment to a cube, at least one point of a triangle shall be in its spatial area.

**Figure 4:**
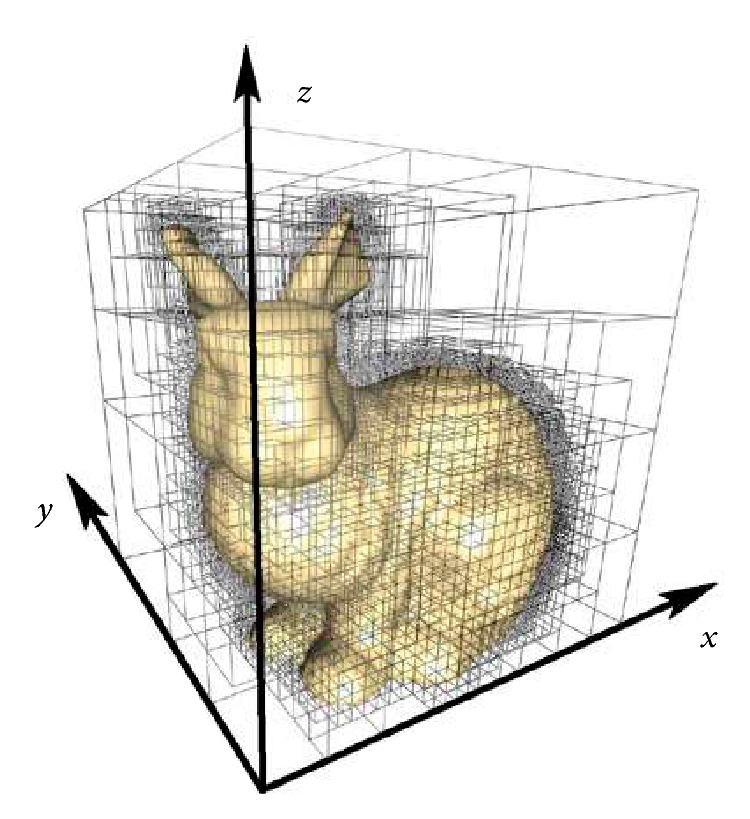
The spatial division employed in the octree hierarchical cubes.

Every eight neighbouring cubes are encapsulated in a bounding box with a double-length edge; such boxes are then encapsulated in another bounding box and so forth. If a cube does not have an assigned triangle, it is completely removed, similarly to a bounding box with no child cube. If a bounding box has only one child, it is substituted by that single child. The result is a tree hierarchical structure (Figure 4).

When testing the 3D model intersection, we start at the top-level bounding box, checking the intersection; if a ray crosses, we check the intersection with the 8 subboxes and so on. Using this approach, we finally reach the cubes at the lowest levels, which are intersected by the ray. Only the triangles belonging to these cubes are tested for intersection. The method distinctly decreases the number of tested triangles, exerting a positive effect on the image mapping performance.

As the algorithm computational time depends on multiple parameters, including, for example, the complexity of a particular 3D model, its resolution, thermal image capturing directions, and the order of the points stored in the memory, it is impossible to estimate the computational effort.

**Figure 5:**
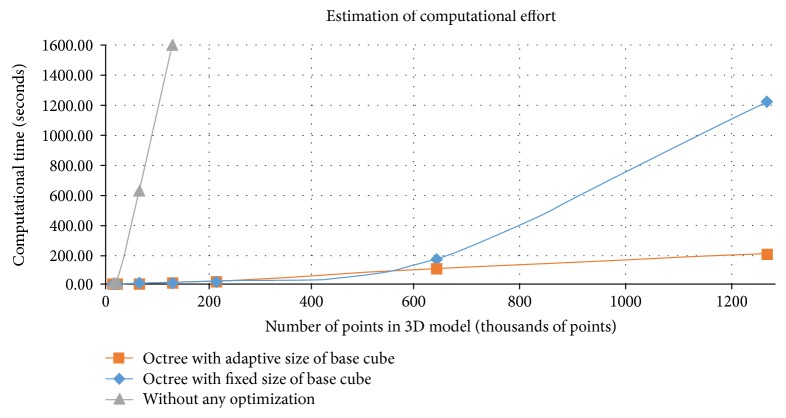
A rough estimation of the optimization performance.

To obtain a rough estimate of the optimization performance, we conducted an experiment where an object was scanned by means of a 3D scanner and a thermal imager in exactly the same manner but at different resolutions. The scanned area corresponded to 100 × 100 mm, with the fixed resolution of 64 points per mm in one axis and the variable resolution of from 0.2 to 20 points in the other. As a result, the number of points fluctuated between 14 thousand and 2.5 million. The results are presented in Figure 5. Here, the grey line indicates the performance without optimization, which grows rapidly even when the resolution still remains very low. The blue line shows the performance in the condition where the octree cube size fixed at 5 mm, a solution linear from the beginning but also exhibiting the tendency towards fast growth with the increasing number of points. The orange line then represents the optimization performance in the scenario of the octree tube adapted according to the average distance between the neighbouring points; this configuration has approximately linear characteristics, pointing to the fact that octree optimization reduces the computational complexity.

## 3. Results

The result of the data fusion method described above lies in a 3D point cloud or a mesh in the same form as that captured by the 3D scanner, enhanced through the thermal information linked with each point of the digital model.

The experiments showed that combining the 3D spatial and thermal data will yield new diagnostic outcomes unavailable with the 3D scanner and thermal imager used separately.

The algorithms were verified in detailed high-resolution meshes captured via RoScan, a robotic 3D scanner able to provide 3D models having a resolution better than 0.1 mm [8, 26, 27]. The thermal images were taken using a LWIR thermal camera Xenics GOBI1954 with the resolution of 384 × 288 pixels, pixel pitch of 25 *μ*m, and spectral response in the wavelength range of 8–14 *μ*m. To establish a computational unit, we employed a desktop computer having an Intel Core i7-4790K processor at 4.00 GHz; 32 GB RAM; and an NVIDIA GeForce GTX 970 GPU.

Due to the octree optimization, the data fusion was quick: the 3D models with 500,000 points merged with the 10 thermal images in only 27 seconds. The resulting data were conveyed in the standard PLY format [28, 29], facilitating the import to multiple 3D analysing software tools; in our experiments, the CloudCompare opensource software was used [30].

**Figure 6:**
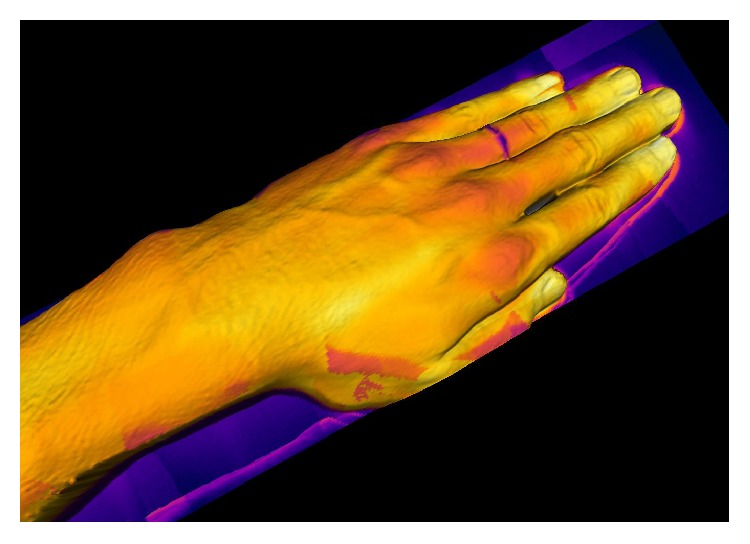
A temperature-mapped 3D model of a hand in the physiological condition. Higher temperatures are observable in the vicinity of the vessels and veins, while lower ones can be located around the joints. The high thermal conductivity of the ring cools the element down, even when put on the finger.

The screenshots from the temperature-mapped 3D models are shown in the related images.  Figure 6 introduces a high-density 3D model of a hand in the physiological condition, with the thermal 3D image displaying even the tiniest details.

**Figure 7:**
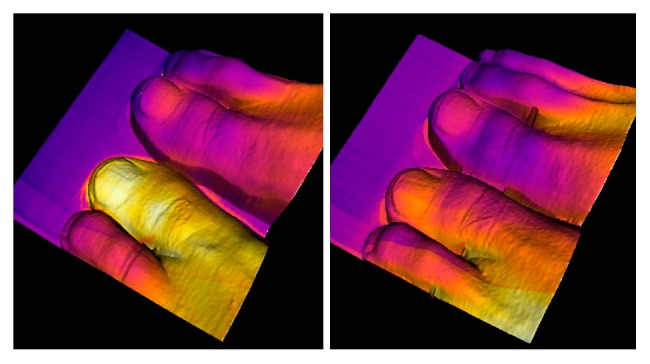
(a) A stubbed toe captured 2 hours after the injury; a precise 3D scan enables us to measure the inflamed area and the swelling-induced volume increase. (b) The same scene 74 hours after the injury; the volume and temperature of the toe have decreased.

Figure 7 presents an inflamed toe after the injury and following the recovery. The injury induced merely light pain, and no other symptoms were observed. A significant temperature increment of 5.12°C is visible in the 3D scan of the afflicted toe; this bodily part also exhibited the volume increment of 5%. Seventy-four hours later, after the recovery, no symptoms or pain was observed; however, the increased temperature was still present in the toe, indicating that the subject had not fully recovered by then. In this context, let us note that the inflammation appears to be determinable and measurable in its very roots, *before* becoming painful. This finding can benefit, for example, top-class athletes and other sportsmen in their efforts to prevent injuries.

**Figure 8:**
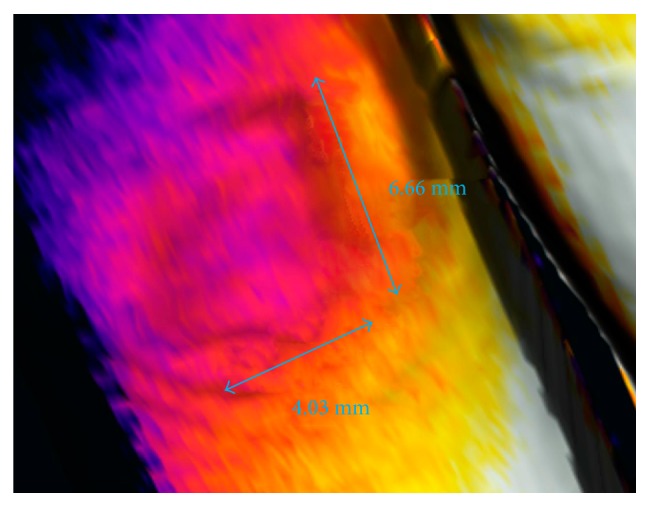
Measuring the dimensions of a glass shard encapsulated in the inner tissue of the finger, unobservable via traditional methods.

Figure 8 demonstrates the ability to measure objects inside the inner tissue, which are otherwise not observable or measurable via traditional approaches. The subject informed us of the injury approximately 2 months ago, and he mentioned an unusual feeling perceived when touching a hard surface with the afflicted finger. The suspected cause consisted in an encapsulated glass shard of unknown dimensions. Although this location could be examined via MRI or CT, these methods are too expensive if employed for the given purpose. Our approach, then, was significantly cheaper while providing the same information; the thermal data served towards defining the boundaries of the encapsulated shard, and the 3D model facilitated precise measurement of the item's dimensions.

It has to be stressed that all the diagnostic information acquired in the cases displayed in Figures 7 and 8, that is, the average temperature and dimensions of the selected region, would not have been available without merging the thermal images and the 3D model. This fact then aptly demonstrates the benefits of the proposed technique.

## 4. Discussion

The described method to merge sets of 2D thermal images with a digital 3D model appears to contribute new diagnostic data unobtainable via traditional methods or through using thermal imaging or 3D scanning separately. The present paper characterizes an algorithm for a general 3D model and images, regardless of the data format. This approach allows us to employ the algorithms also in other research applications or medical diagnostic tools.

Considering its principles, the method is suitable for rendering high-density point clouds or detailed meshes at a high resolution; conversely, the technique cannot be conveniently utilized in large objects with sparse points.

The benefits of creating 3D thermal models have already been demonstrated on practical experiments with injured subjects. The findings published within article [31] show that thermal imagers constitute a useful, versatile diagnostic tool which, when combined with 3D scanners, significantly increases the amount of data to facilitate precise diagnostics or monitoring.

This method finds use within not only the medical but also the technological domain: the data fusion between thermal imagers and 3D scanners will bring numerous advantages in, for example, robotic rescue systems [32, 33], where the potential of the technique may be exploited for augmented reality [18].

## Additional Points

*Main Messages*. (i) 3D thermal imaging facilitates quantification, a step not performable with 2D thermal imaging. (ii) Combining 3D and thermal imaging yields more useful diagnostic data. (iii) Practical experiments on injured subjects display possible target application cases. (iv) A general recipe for fusing a thermal image and a 3D model is proposed, offering broad usability with common data types.
